# Expanding the mutational spectrum in Johanson‐Blizzard syndrome: identification of whole exon deletions and duplications in the *UBR1* gene by multiplex ligation‐dependent probe amplification analysis

**DOI:** 10.1002/mgg3.319

**Published:** 2017-07-31

**Authors:** Maja Sukalo, Eva Schäflein, Ina Schanze, David B. Everman, Nima Rezaei, Jesús Argente, Isabel Lorda‐Sanchez, Charu Deshpande, Tsutomu Takahashi, Alexander Kleger, Martin Zenker

**Affiliations:** ^1^ Institute of Human Genetics University Hospital Magdeburg Magdeburg Germany; ^2^ Institute of Human Genetics University of Erlangen‐Nuremberg Erlangen Germany; ^3^ Department of Psychosomatic Medicine and Psychotherapy University Hospital Rechts der Isar, Technische Universität München Munich Germany; ^4^ Greenwood Genetic Center Greenwood South Carolina; ^5^ Research Center for Immunodeficiencies Children's Medical Center Tehran University of Medical Sciences Tehran Iran; ^6^ Network of Immunity in Infection, Malignancy and Autoimmunity (NIIMA) Universal Scientific Education and Research Network (USERN) Sheffield UK; ^7^ Departments of Endocrinology and Pediatrics and Instituto de Investigación La Princesa Hospital Infantil Universitario Niño Jesús Madrid Spain; ^8^ Department of Pediatrics Universidad Autónoma de Madrid Madrid Spain; ^9^ Centro de Investigación Biomédica en Red Fisiopatología de la Obesidad y Nutrición (CIBEROBN) Instituto de Salud Carlos III Madrid Spain; ^10^ Department of Genetics IIS‐Fundación Jiménez Díaz UAM CIBERER Madrid Spain; ^11^ Clinical Genetics Guy's Hospital London UK; ^12^ Department of Pediatrics Akita University Graduate School of Medicine Akita Japan; ^13^ Department of Internal Medicine I University Medical Center Ulm Ulm Germany

**Keywords:** autosomal recessive, Johanson‐Blizzard syndrome, MLPA, multiplex ligation‐dependent probe amplification, UBR1

## Abstract

**Background:**

Johanson‐Blizzard syndrome (JBS, MIM #243800) is a very rare autosomal recessive disorder characterized by exocrine pancreatic insufficiency, nasal wing hypoplasia, hypodontia, and other abnormalities. JBS is caused by mutations of the *UBR1* gene (MIM *605981), encoding a ubiquitin ligase of the N‐end rule pathway.

**Methods:**

Molecular findings in a total of 65 unrelated patients with a clinical diagnosis of JBS who were previously screened for UBR1 mutations by Sanger sequencing were reviewed and cases lacking a disease‐causing UBR1 mutation on either one or both alleles were included in this study. In order to discover mutations that are not detectable by Sanger sequencing, we designed a probe set for multiplex ligation‐dependent probe amplification (MLPA) analysis of the *UBR1* gene and analyzed the copy number status of all 47 *UBR1* exons.

**Results:**

Our previous studies using Sanger sequencing could detect mutations in 93.1% of 130 disease‐associated *UBR1* alleles. Six patients with a highly suggestive clinical diagnosis of JBS and unsolved genotype were included in this study. MLPA analysis detected six alleles harboring exon deletions/duplications, thereby raising the mutation detection rate in the entire cohort to 97.7% (127/130 alleles).

**Conclusion:**

We conclude that single or multi‐exon deletions or duplications account for a substantial proportion of JBS‐associated *UBR1* mutations.

Johanson‐Blizzard syndrome (JBS, MIM #243800) is a clinically distinct, autosomal recessively inherited congenital malformation syndrome. The major clinical hallmarks of this multisystem disorder are exocrine pancreatic insufficiency (EPI), nasal wing hypo‐/aplasia, and oligodontia of permanent teeth. Hearing impairment, scalp defects, cognitive impairment of variable degree, short stature, hypothyroidism, microcephaly, intrauterine growth restriction, congenital heart defects, urogenital and anorectal malformations, renal anomalies, and diabetes with onset during adolescence are additional features of the syndrome (Johanson and Blizzard 1971; Zenker 2008; Sukalo et al. 2014). More than 100 cases of this rare disease have been reported. Homozygous and compound heterozygous mutations of the *UBR1* gene (MIM *605981, NM_174916.2) are the molecular basis of JBS (Zenker et al. 2005). The human *UBR1* gene contains 47 exons that encode a ubiquitin ligase of the N‐end rule pathway, an evolutionary conserved and ubiquitously expressed intracellular proteolytic pathway involved in ubiquitin‐mediated degradation of many proteins (Varshavsky 1996).

Based on the large cohort consisting of 61 patients from 50 families that was published by our group in 2014 (Sukalo et al. 2014), EPI with onset in infancy, nasal wing hypo‐/aplasia, and oligodontia of permanent teeth emerged as consistent phenotypic signs and useful major clinical criteria for JBS. Four additional features that are present in 79% to 21% of cases can specifically support the clinical diagnosis of JBS as minor criteria: sensorineural deafness, scalp defect, hypothyroidism, and imperforate anus (Table 1). During the last 2 years we were able to investigate additional 15 patients from 15 families. Thus, 65 unrelated JBS‐affected families (by November 2016) were studied at our institution, of which a majority of 59 families has already been published by Sukalo (2016). The study was approved by the Ethics Board of the Medical Faculty of the University of Erlangen. Informed consent was obtained from all patients or parents. In this clinically well‐defined patient cohort, we could detect mutations in 93.1% of all *UBR1* alleles (121 of 130) by Sanger sequencing of coding exons and flanking intronic regions (±20 bp). Mutations included 37 nonsense, 30 splice site, 32 missense, and 19 frameshift mutations, as well as three small in frame deletions. In nine *UBR1* alleles no mutations were detected by Sanger sequencing.

**Table 1:** Johanson‐Blizzard syndrome patients investigated by Sanger sequencing and MLPA analysis

Patient	Mutations	Gender	Age	Exocrine pancreatic insufficiency^a^	Nasal wings gestalt^a^	Oligodontia of permanent teeth^a^	Hearing impairment^b^	Scalp defect^b^	Short stature (P < 3)	Cognitive impairment	Hypothyroidism^b^	Microcephaly (P < 3)	Congenital heart defect	Intrauterine growth retardation (P < 3)	Imperforate anus^b^	Genital malformation	Renal anomalies	Diabetes (age of onset/diagnosis)	Parental consanguinity	Reference
1	p.Tyr1508*+c.(4835+1_4836‐1)_(*1_?)del	F	9.5y	+	A	+	+	+	−	NS	+	−	−	−	−	−	−	−	−	1
2	c.(1281+1_1282‐1)_(1439+1_1440‐1)del+c.(1281+1_1282‐1)_(1439+1_1440‐1)del	F	2.5 m	+	A	na	+	−	na	na	+	na	ASD	−	−	−	−	na	+	−
3	c.(2739+1_2740‐1)_(3209+1_3210‐1)del+c.(3209+1_3210‐1)_(3415+1_3416‐1)dup	M	8 m	+	A	na	+	+	+	na	−	na	ASD	−	−	−	−	−	−	−
4	c.(3659+1_3660‐1)_(3757+1_3758‐1)del+p.Leu1597Arg	F	4.3y	+	A	na	+	+	+	na	−	na	−	+	−	−	−	−	−	−
5	p.Ala563Asp+(?)	F	37y	+	H	+	+	−	+	BL	−	na	−	+	−	−	−	(18)	−	1
6	(?)+(?)	M	5.9y	+	H	+	+	+	+	NS	+	−	−	−	−	−	−	−	+	2
% in the entire JBS cohort (79 patients)				100	100	100	79	64	62	62	41	37	28	26	21	19	12	8		

F, female; M, male; y, year(s); m, month(s); +, present; −, not present; A, aplasia; H, hypoplasia; na, no data available; NS, present but not specified; BL, borderline intellectual disability; ASD, atrial septal defect; P < 3, percentile below 3rd (according to growth charts from World Health Organisation and Robert Koch Institute). Mutation nomenclature refers to GenBank reference sequence NM_174916.2 and GRCh37/hg19. Nucleotide numbering reflects cDNA numbering with + 1 corresponding to the A of the ATG translation initiation codon in the reference sequence, according to journal guidelines (www.hgvs.org/mutnomen and varnomen.hgvs.org/recommendations/DNA/variant/deletion). The initiation codon is codon 1. References: (1) Sukalo et al. 2014; (2) Takahashi et al. 2004.

a Major clinical criteria of JBS.

b Minor, but specific clinical criteria of JBS.

In this cohort, three unrelated patients with a highly suggestive clinical diagnosis of JBS remained without a detectable *UBR1* mutation, and in three additional affected individuals a *UBR1* mutation could be detected in only one allele by conventional Sanger sequencing of all coding exons and flanking intronic regions. However, using such an approach it is not possible to detect mutations of the promoter region, intronic mutations that are not located close to the splice sites, larger genomic deletions and duplications, or rearrangements of the gene. In order to increase the sensitivity of the mutation screening, we chose a self‐designed multiplex ligation‐dependent probe amplification (MLPA) assay for the *UBR1* gene. Using such an MLPA approach facilitates relative quantification of nucleic acid fragments. Hence, it is possible to detect copy number changes caused by duplications and deletions of the genomic target region like the exons of a gene. MLPA based detection of exon deletions and duplications was first described by Schouten et al. (2002). The underlying mechanism is the quantification of PCR amplification products from variably‐sized oligonucleotides that are generated by ligation of pairs of specific probes after binding to complementary DNA targets. Each half‐probe contains a universal primer binding site (PBS, universal 5′ PBS: GGGTTCCCTAAGGGTTGGA, universal 3′ PBS: TCTAGATTGGATCTTGCTGGCAC) and a unique hybridizing sequence. We designed unique oligonucleotide probes (Table S1) for each of the 47 exons of *UBR1* according to the protocol by MRC‐Holland (https://www.mlpa.com) (Stern et al. 2004). Synthetic oligonucleotides were purchased from metabion GmbH (Planegg‐Martinsried, Germany). Hybridizing sequences were designed avoiding to overlap with the targets of oligonucleotide primers used for PCR and Sanger sequencing, in order to generate two fully independent analysis methods. Generally, the unique hybridizing target sequence was located within the respective exon. Additionally, we avoided placing the probes on targets containing known polymorphisms. The 47 synthetic *UBR1* probe pairs were divided into five probe mixes according to the sizes of the expected products, which ranged between 88 and 128 nucleotides (Table S2). For each analysis, at least three control DNAs were included. These reference samples tested negative for copy number abnormalities of >50 kb at the *UBR1* locus using a CytoScan HD Array (Affymetrix, Santa Clara, CA). Denaturation, hybridization, ligation, and MLPA‐PCR were performed in an iCycler (Bio‐Rad Laboratories Inc., Hercules, CA) (Fig. S1). SALSA MLPA reagents and the P200 Human DNA reference‐1 probemix were used (MRC‐Holland, Amsterdam, The Netherlands). GeneScan 500 LIZ dye Size Standard and POP‐7 Performance Optimized Polymer were used for analysis on the 3500xL Genetic AnalyZer (Thermo Fisher Scientific Inc., Waltham, MA). Analysis of the electropherograms was performed with JSI Sequence Pilot software Version 4.2.1 (JSI medical systems GmbH, Ettenheim, Germany). Detailed description of the applied method can be found in the Supporting Information. All *UBR1* mutations that were detected in our lab can be found at the Leiden Open Variation Database (LOVD, http://databases.lovd.nl/shared/variants/UBR1).

Patient 1 (Fig. 1A,B) was originally published by Sukalo et al. in 2014 (patient JBS‐26.1). At that time, only a maternally inherited heterozygous nonsense mutation of the *UBR1* gene (c.4524T>A, p.Tyr1508*) was identified in the patient (Fig. 1D). The clinical diagnosis of JBS was based on the presence of the three major criteria, namely nasal wing hypoplasia, EPI, and hypodontia of permanent teeth, supported by the specific minor criteria hearing impairment, scalp defects, and hypothyroidism (Table 1). MLPA analysis revealed a deletion of exons 45, 46, and 47 (c.(4835+1_4836‐1)_(*1_?)del), as illustrated in Figure 1E. The deletion was not present in the mother and is therefore assumed to be located on the paternal allele, but verification of this assumption was not possible due to lack of a paternal DNA sample.

**Figure 1:**
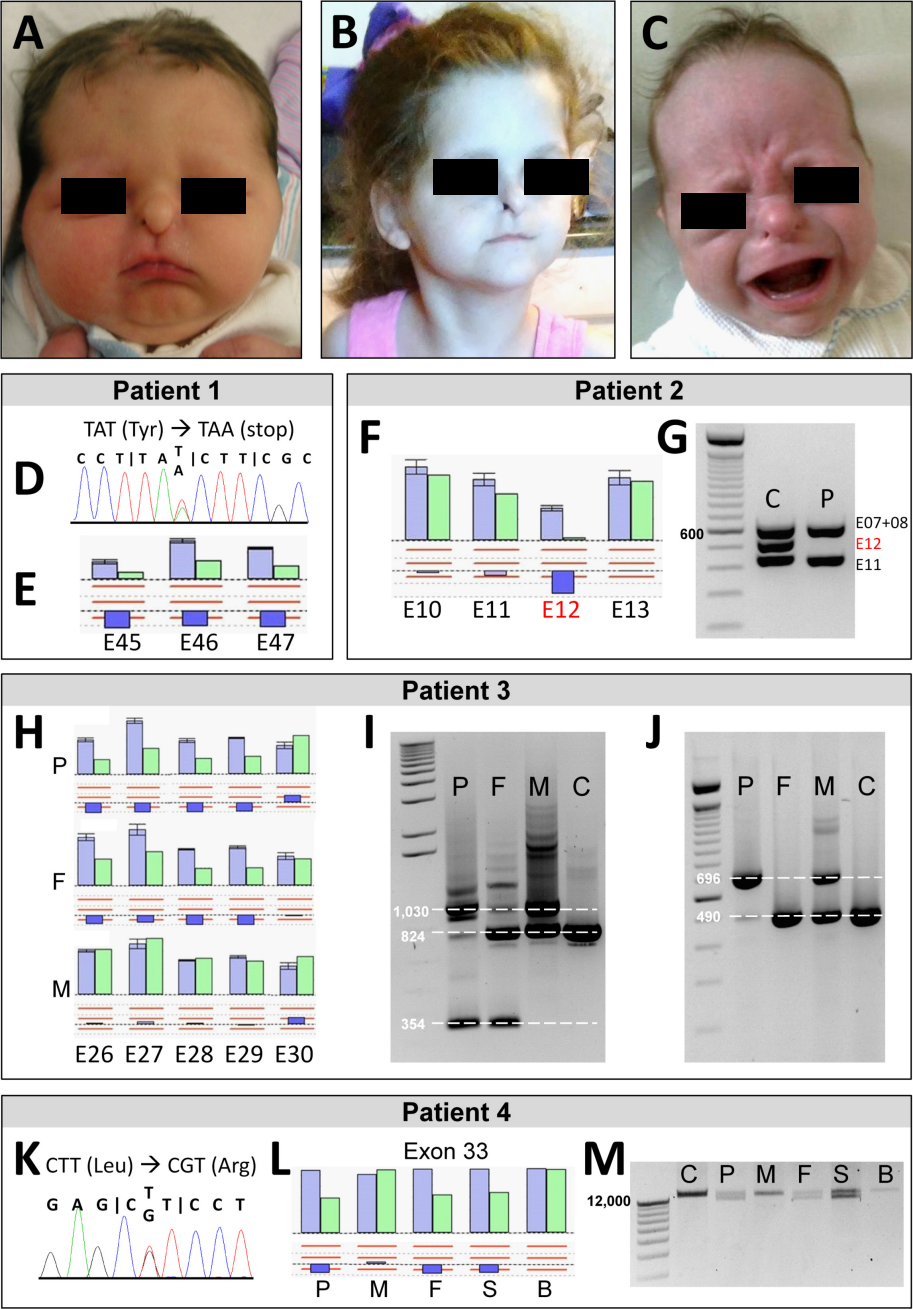
Analysis of whole exon deletions and duplications in the *UBR1* gene (NM_174916.2). (A–C) The patients with whole exon deletions/duplications in *UBR1* show typical facial findings of JBS. (A) Patient 1 at age 2 days, showing aplasia of the nasal wings. (B) Patient 1 at age 9 years and 6 months, with aplastic alae nasi and frontal upsweep of the hair. (C) Patient 3 at age 8 months, with aplasia of the nasal wings and frontal upsweep of the hair. (D) Electropherogram showing the c.4524T>A (p.Tyr1508*) nonsense mutation in patient 1, identified by Sanger sequencing of exon 41. (E) Bar chart representing the deletion of exons 45, 46, and 47 detected by MLPA analysis of genomic DNA from patient 1. (F) Results of MLPA analysis in patient 2 showing a homozygous deletion of exon 12 and normal results for the adjacent exons 10, 11, and 13. (G) Multiplex PCR from the same patient showing no band at the expected size (547 bp) for the PCR product containing exon 12 in the patient's sample, while control sample shows expected PCR products. (H) MLPA results for patient 3 and his parents showing segregation of two intragenic copy number changes (paternally inherited multi‐exon deletion and maternally inherited exon 30 duplication). (I) PCR on cDNA from the same family using a primer combination spanning exons 26–30. The small fragment at 354 bp represents the allele lacking four exons in the patient and his father. Wild type allele is seen in the father, mother and control at 824 bp, but not in the patient. An additional band at 1030 bp representing the exon 30 duplication is visible in the samples derived from the patient and his mother. (J) Allele‐specific PCR on cDNA using a forward primer within the paternally inherited deletion. The patient shows only an elongated PCR product representing the duplication allele (696 bp), the mother has a wild type allele (490 bp) and a duplication allele, and the father and control have wild type alleles only. The patient's PCR product was then subjected to Sanger sequencing confirming the exon 30 duplication (data not shown). (K) Electropherogram showing the c.4790T>G (p.Leu1597Arg) missense mutation in patient 4, generated by Sanger sequencing of exon 44. This heterozygous substitution was also detected in the mother and in the unaffected brother. (L) MLPA results for patient 4 and her family showing heterozygous deletion of exon 33 in the patient, her father, and her unaffected sister. (M) Long‐range PCR using a forward primer located upstream of exon 32 and a reverse primer downstream of exon 34. Expected size of wild type allele is 13,043 bp. The family members that were found to have a heterozygous deletion of exon 33 by MLPA consistently showed two bands in gel electrophoresis, representing the wild type allele and a shortened (mutated) allele. E, exon; C, control; P, patient; F, father; M, mother; S, sister, B, brother.

The phenotype of patient 2, who was born to consanguineous parents, was considered typical of JBS, based on the presence of EPI and aplastic alae nasi, together with sensorineural hearing loss and hypothyroidism (Table 1). The status of the secondary teeth could not be assessed clinically due to the patient's young age. Sanger sequencing did not reveal any mutation of the *UBR1* gene. However, no reproducible PCR product could be obtained for exon 12 indicating that genetic integrity of this exon might be disturbed. Consequently, we performed MLPA analysis for this patient, confirming a homozygous deletion of exon 12 (Fig. 1F). To verify the deletion of exon 12 with an independent method, multiplex PCR was performed (Fig. 1G). We could confirm the homozygous deletion of exon 12, as the patient consistently showed no band at the expected size for the product from exon 12 (547 bp). Unfortunately, verification of the heterozygous exon 12 deletion by MLPA on parental samples was not possible.

The clinical diagnosis of JBS in patient 3 was based on the presence of EPI and nasal wing aplasia, together with severe hearing loss and an occipital scalp defect (Table 1 and Fig. 1C). Again, the status of the permanent teeth could not be assessed due to the young age of the patient and Sanger sequencing of the *UBR1* gene did not reveal any mutation. Subsequent MLPA analysis identified a heterozygous deletion of exons 26–29 of paternal origin (c.(2739+1_2740‐1)_(3209+1_3210‐1)del), while the probe for exon 30 indicated an increased dosage in the patient and in the maternal sample (Fig. 1H). PCR primers were designed spanning exons 26‐30 (forward primer located within exon 25 and reverse primer located at the exon‐exon boundary between exons 30 and 31). Amplifying cDNA from the patient and his parents, a shorter fragment resulting from the exon 26‐29 deletion could be demonstrated in the patient and his father, as well as an elongated fragment of ~1000 bp in the patient and his mother, representing the duplication of exon 30 (Fig. 1I). Next, an allele specific PCR in which only the wild type and the maternally inherited alleles were amplified was performed on the patient's cDNA (Fig. 1J). This was accomplished by designing a forward primer binding in the region that is deleted on the paternal allele. Sequencing of this elongated fragment confirmed the tandem duplication of exon 30 in the cDNA (c.(3209+1_3210‐1)_(3415+1_3416‐1)dup).

The diagnosis of patient 4 was based on EPI and aplasia of nasal wings. Additionally, she presented with sensorineural hearing loss and parietal and occipital scalp defects. Once again, the status of the permanent teeth could not be assessed due to the young age of the patient. We detected the missense mutation p.Leu1597Arg (c.4790T>G) in exon 44 in one allele of this patient by Sanger sequencing (Fig. 1K). In silico prediction by various online tools (MutPred, PolyPhen‐2, MutationAssessor, SIFT, MutationTaster) consistently rates this amino acid substitution to be pathogenic (Table S3). The leucine residue at position 1597 of the UBR1 protein is highly conserved among different species (Fig. S2). The same heterozygous mutation was detected in her mother and an unaffected brother. In the second allele, a deletion of exon 33 (c.(3659+1_3660‐1)_(3757+1_3758‐1)del) was detected by MLPA analysis in the patient, her father and her unaffected sister (Fig. 1L). Deletion of exon 33 was additionally verified by long‐range PCR of the family's DNA samples using a forward primer located upstream of exon 32 and a reverse primer downstream of exon 34 (Fig. 1M).

In patient 5 we were previously able to detect a heterozygous missense mutation (c.1688C>A, p.Ala563Asp) by Sanger sequencing, but no mutation in the second allele could be detected (patient 24.1 in (Sukalo et al. 2014)). However, by investigating the point mutation in cDNA from leukocytes, significant underrepresentation of the second allele (wild‐type for the mutation) was demonstrated (Sukalo et al. 2014). This suggested a reduced expression or increased degradation of the *UBR1* allele that does not harbor the missense mutation, probably due to a mutation that could not be detected by conventional sequencing. This patient had a clinical diagnosis of JBS based on the combination of EPI, nasal wing hypoplasia, oligodontia of permanent teeth, and progressive hearing loss (Table 1). MLPA analysis in this patient showed normal results, thus excluding that the mutation in the second *UBR1* allele, which is indirectly demonstrated by the results of cDNA analysis, is a whole exon deletion or duplication. Nevertheless, the molecular findings in this patient suggest that a *UBR1* defect is the underlying reason for her JBS phenotype. A mutation in the promoter or intronic regions as well as a larger genomic rearrangement could be the explanation.

Patient 6 was clinically described by Takahashi et al. (2004). He showed all three major criteria for JBS, namely EPI, hypoplasia of alae nasi, and hypodontia of permanent teeth, accompanied by hearing impairment, a parietal scalp defect, and hypothyroidism (Table 1). A parental first cousin marriage was reported. Sanger sequencing did not reveal any pathogenic changes in the *UBR1* gene. MLPA analysis of the 47 coding exons of the *UBR1* gene was also negative in this patient. Moreover, a SNP array (Affymetrix) detected no homozygosity at the *UBR1* locus and by western Blot analysis no reduction in UBR1 protein expression could be demonstrated (data not shown).

Taken together, MLPA‐based screening detected five different single or multi‐exon deletions/duplications of *UBR1*, thereby expanding the mutational spectrum of the *UBR1* gene. The six unrelated patients investigated in this study had a typical JBS phenotype but no *UBR1* point mutation detectable by Sanger sequencing in either one allele (n = 3) or both alleles (n = 3), together representing a total of nine JBS‐associated *UBR1* alleles without a detectable mutation. MLPA analysis was able to identify the underlying mutation in six of nine previously unsolved alleles. Considering the entire cohort of 65 unrelated patients (including patients published by Sukalo et al. 2014 as well as new unpublished data), the mutation detection rate by Sanger sequencing alone reached 93.1% (121 out of 130 disease‐associated alleles). Whole exon deletions and duplications detectable by MLPA accounted for 4.6% of disease‐associated alleles, thus increasing the mutation detection rate in the *UBR1* gene in a clinically well‐characterized cohort with a JBS phenotype to 97.7%. Such a proportion of larger exonic deletions/duplications is not unusual in the mutational spectrum of genes for autosomal recessive disorders (see Phenylalanine Hydroxylase Deficiency, Shwachman‐Diamond Syndrome, and Kindler Syndrome in GeneReviews^®^ (Pagon et al. 1993–2016)). Nevertheless, our cohort of JBS patients might have a bias toward rare whole exon deletions/duplications, as some samples were sent to us after conventional Sanger sequencing failed to detect biallelic mutations.

The remaining 2.3% (3 disease‐associated alleles in two unrelated patients) that were not solved may be explained by mutations in the promoter region, intronic mutations that are not close to the splice sites or larger genomic rearrangements (e.g., inversions) of the *UBR1* gene, which cannot be captured by either of the applied methods. This is probably true for patient 5 in whom we have indirect evidence for the existence of a genetic change in the second *UBR1* allele through the demonstration of decreased mRNA expression (Sukalo et al. 2014). In contrast, for patient 6 we also have to consider that a mutation or mutations in another gene might be responsible for the disorder, as all molecular investigations provided no evidence of involvement of the *UBR1* gene (no mutation identified by sequencing and MLPA, heterozygosity at the *UBR1* locus in the presence of parental consanguinity, normal UBR1 protein expression in leukocytes). However, if there is indeed a second gene associated with JBS, its patient‐wide contribution is very small.

In conclusion, we recommend deletion/duplication analysis of the *UBR1* gene in patients with a distinct JBS phenotype, if point mutation screening does not reveal a *UBR1* mutation in one or both alleles.

## Conflict of Interest

The authors declare no conflict of interest.

## Supporting information


**Appendix S1.** Material and methods.
**Table S1.** MLPA probes for analysis of the *UBR1* gene.
**Table S2.** *UBR1* probemixes (for deletion/duplication screening).
**Table S3.** Prediction of pathogenicity of the *UBR1* missense variant p.Leu1597Arg (c.4790T>G) using various online prediction tools.
**Figure S1.** Schematic overview of an MLPA procedure.
**Figure S2.** Multiple protein alignment of human UBR1 and its orthologues around position 1597.
