# Supplementary material for: Expanding the mutational spectrum in Johanson‐Blizzard syndrome: identification of whole exon deletions and duplications in the UBR1 gene by multiplex ligation‐dependent probe amplification analysis
Source: Mol Genet Genomic Med. 2017 Jul 31;5(6):774–80. doi: 10.1002/mgg3.319 (PMC5702574; doi:10.1002/mgg3.319)
Supplement: Supplementary file 1 — Appendix S1. Material and methods. Table S1. MLPA probes for analysis of the UBR1 gene. Table S2. UBR1 probemixes (for deletion/duplication screening). Table S3. Prediction of pathogenicity of the UBR1 missense variant p.Leu1597Arg (c.4790T>G) using various online prediction tools. Figure S1. Schematic overview of an MLPA procedure. Figure S2. Multiple protein alignment of human UBR1 and its orthologues around position 1597. [file MGG3-5-774-s001.docx]

**Expanding the Mutational Spectrum in Johanson-Blizzard Syndrome: Identification of Whole Exon Deletions and Duplications in the *UBR1* Gene by Multiplex Ligation-Dependent Probe Amplification (MLPA) Analysis**

Maja Sukalo^1^, Eva Schäflein^2,3^, Ina Schanze^1^, David B. Everman^4^, Nima Rezaei^5,6^, Jesús Argente^7,8,9^, Isabel Lorda-Sanchez^10^, Charu Deshpande^11^, Tsutomu Takahashi^12^, Alexander Kleger^13^, Martin Zenker^1^

^1^Institute of Human Genetics, University Hospital Magdeburg, Magdeburg, Germany. ^2^Institute of Human Genetics, University of Erlangen-Nuremberg, Erlangen, Germany. ^3^Department of Psychosomatic Medicine and Psychotherapy, University Hospital Rechts der Isar, Technische Universität München, Munich, Germany. ^4^Greenwood Genetic Center, Greenwood, South Carolina, USA. ^5^Research Center for Immunodeficiencies, Children's Medical Center, Tehran University of Medical Sciences, Tehran, Iran. ^6^Network of Immunity in Infection, Malignancy and Autoimmunity (NIIMA), Universal Scientific Education and Research Network (USERN), Sheffield, UK. ^7^Departments of Endocrinology and Pediatrics and Instituto de Investigación La Princesa, Hospital Infantil Universitario Niño Jesús, Madrid, Spain. ^8^Department of Pediatrics, Universidad Autónoma de Madrid, Madrid, Spain. ^9^Centro de Investigación Biomédica en Red Fisiopatología de la Obesidad y Nutrición (CIBEROBN), Instituto de Salud Carlos III, Madrid, Spain. ^10^Department of Genetics, IIS-Fundación Jiménez Díaz UAM, CIBERER, Madrid, Spain. ^11^Clinical Genetics, Guy’s Hospital, London, UK. ^12^Department of Pediatrics, Akita University Graduate School of Medicine, Akita, Japan. ^13^Department of Internal Medicine I, University Medical Center Ulm, Ulm, Germany.

**Supporting Material and Methods**

All DNA samples (patients and controls) were diluted in ultra-pure H_2_O to a final concentration of 20 ng/µl, equaling a total amount of 100 ng DNA in each reaction with a volume of 5 µl. If not indicated otherwise, the reagents were obtained from MRC-Holland (Amsterdam, The Netherlands). Steps 1 to 4 were carried out in an iCycler (Bio-Rad Laboratories Inc., Hercules, USA). Self-designed oligonucleotide probes were ordered at metabion GmbH (Planegg-Martinsried, Germany). Synthetic probe design was performed according to the protocol by MRC-Holland (https://www.mlpa.com). An overview of the MLPA procedure is schematically displayed in Figure S1.

**Step 1:** The DNA solution was heated for 5 min at 98°C for denaturation of the double strands.

**Step 2:** For hybridization, 1 µl SALSA MLPA P200 Human DNA reference-1 probemix, 0.5 µl UBR1 self-designed probemix (Supp. Tab. S1, and S2), and 1.5 µl SALSA MLPA buffer were added per sample. Samples were incubated at 95°C for 1 min followed by 60°C for 16-18 hours of hybridization.

**Step 3:** For ligation of the half-probes, 3 µl Ligase buffer A, 3 µl Ligase buffer B, 25 µl ultra-pure H_2_O, and 1 µl ligase per sample were added. After 15 min at 54°C, half-probes that had already hybridized to the DNA targets and were adjacent to each other, were ligated to form a probe of the desired length. Afterwards, the ligase was inactivated for 5 min at 98°C.

**Step 4:** For PCR amplification, 2 µl SALSA PCR primer mix (fluorescently labelled universal primers), 7.5 µl ultra-pure H_2_O, and 0.5 µl SALSA polymerase were added to 20 µl of the mix containing the ligated probes. PCR reaction included 35 cycles (denaturation: 95°C for 30 sec, annealing: 60°C for 30 sec, elongation: 72°C for 60 sec) followed by a final elongation (72°C for 20 min) and was cooled down afterwards to 20°C. Exponential amplification allows quantification of exon copy numbers, as read out depends on the amount of hybridized and successfully ligated half-probes.

**Step 5:** The PCR products were diluted 1:3 with ultra-pure H_2_O, then 0.5 µl of the dilution was mixed with 10 µl Hi-Di Formamide and 0.1 µl GeneScan 500 LIZ dye Size Standard (Thermo Fisher Scientific Inc., Waltham, USA). This mixture was heated to 98°C for 2 min and chilled on ice. The samples were injected into a 3500xL Genetic AnalyZer (Thermo Fisher Scientific Inc., Waltham, USA) using the standard program MLPA_POP7xl. Gel electrophoresis within the capillaries of the sequencer separated the probes by length and detection was possible due to the fluorescently labelled primers that were included in the PCR.

**Step 6:** Analysis of the obtained electropherograms was performed with JSI Sequence Pilot software Version 4.2.1 (JSI medical systems GmbH, Ettenheim, Germany).

**Supp. Figure S1:** Schematic overview of an MLPA procedure.


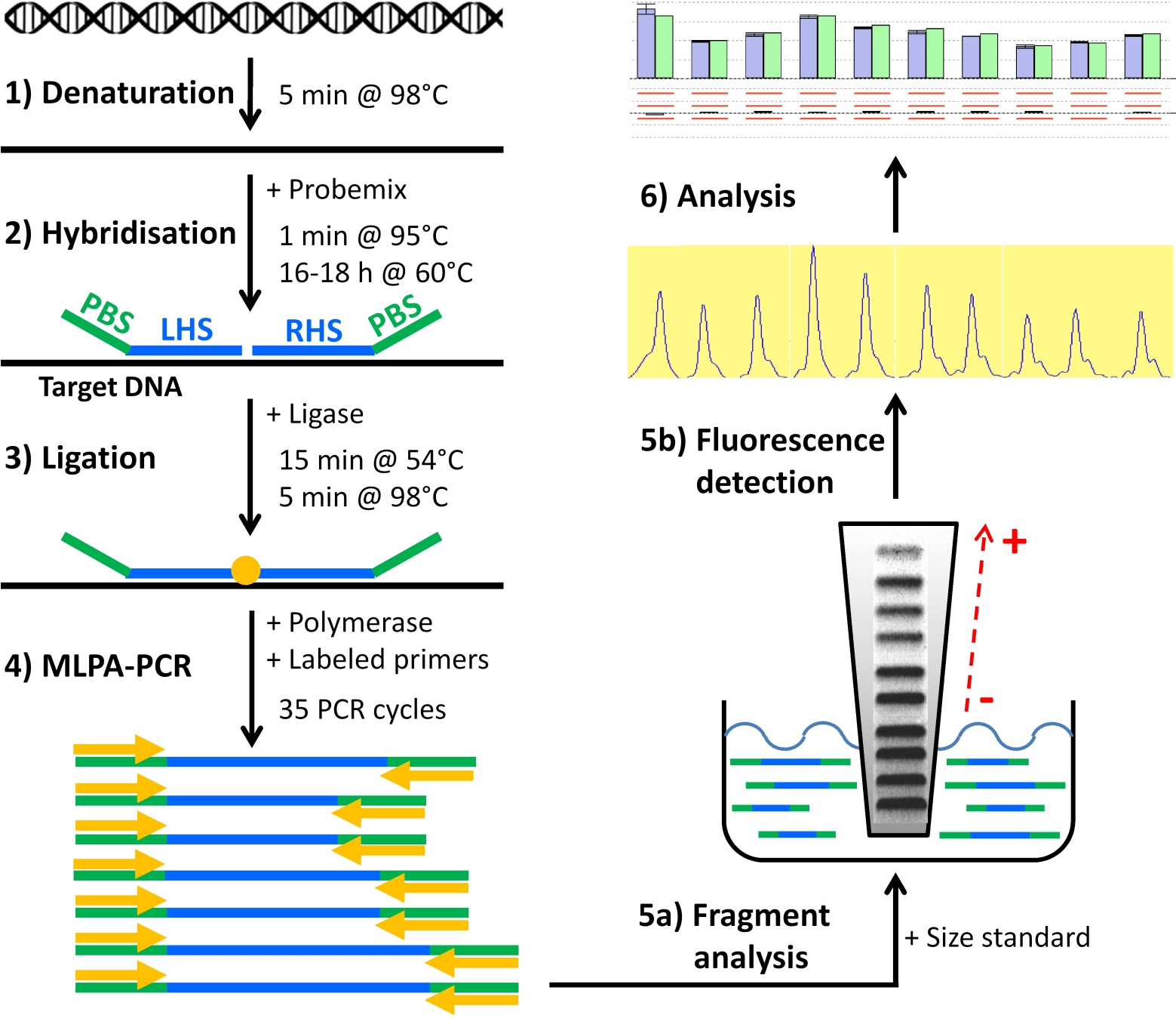


PBS, primer binding sequence; LHS, left hybridizing sequence; RHS, right hybridizing sequence.

**Supp. Table S1:** MLPA probes for analysis of the *UBR1* gene.

| **Exon** | **Sequence (5‘–3‘)** | **Length** | **Mix** |
| --- | --- | --- | --- |
| 1 | CGGACGAGGAGGCTGGAGGTACTGAGAGGATGGAAATC  AGCGCGGAGTTACCCCAGACCCCTCAGCGTCT | 112 nt | A |
| 2 | ATTTGGAGAAGATCCAGATATTTGCTTAGAGAAATTG  AAGCACAGTGGAGCATTTCAGCTTTGTGGGAGGGTTT | 116 nt | A |
| 3 | GGATTGTGCAATTGATCCAACATGTG  TACTCTGTATGGACTGCTTCCAGGACAGTGTT | 100 nt | A |
| 4 | GTGACTGTGGAGACACAGAGGCATGGAAAACTGGCCC  TTTTTGTGTAAATCATGAACCTGGAAGAGCAGGTACTATAAAAGA | 124 nt | A |
| 5 | ctcggcttttttatagAATTCACGCTGTCCGTTGAATG  AAGAGGTAATTGTCCAAGCCAGGAAAATATTTCCTT | 116 nt | B |
| 6 | CCACGTCATATCGTCATATACAGCCTACAAAGAGCTCTTGAC  TGTGAGCTCGCAGAGGCCCAGTTGCATACCACTGCC | 120 nt | A |
| 7 | GTCGTCGGGCTGTTAAAGCGGGAGCTTATG  CTGCTTGCCAGGAAGCAAAGGAAGATATAAAG | 104 nt | A |
| 8 | CTCTCAACATCCACTTCATGTAGAAGTATTACACTCAG  AGATTATGGCTCATCAGAAATTTGCTTTGCGTCTTGGTTC | 120 nt | B |
| 9 | GAGAATCCCTGTCTCATAAGCAGGTTAATGCTTTGGGATG  CAAAGCTTTATAAAGgtaagtagacatttgcttatgctgttt | 124 nt | B |
| 10 | GCCCGTAAGATCCTTCATGAATTG  ATCTTCAGCAGTTTTTTTATGGAGATGGAA | 96 nt | A |
| 11 | CAGTGATGATCATGACCAGAAGTATCTCTATAACTGCAC  TTTCAGTTCAGATGTTTACTGTTCCTACTCGgtatgtat | 120 nt | C |
| 12 | CTTATTGAAGAGCAGAATGTTATCTCTGTCATTACTGAAACTC  TGCTAGAAGTTTTACCTGAGTACTTGGACAGGAACAATA | 124 nt | C |
| 13 | GCAAACCCACAATATGGACAGAAAGATTAAGAATGCAGTTC  CTTGAAGGTTTTCGATCTTTTTTGAAGATTCTTACCTGTATGCAG | 128 nt | A |
| 14 | CAGGTTGGGCAACACATTGAAGTGGATCCTGATTGGGAGG  CTGCCATTGCTATACAGATGCAATTGAAGAATATTTTACTCA | 124 nt | D |
| 15 | ggtatatcttatgtttcctacaagtttgcagatcctgtggag  gtagacttagtaatacatagaacaattttccactcatatttagt | 128 nt | B |
| 16 | GTCTTCATGTACGTTTAAGCAGGCTGGGTG  CTGTTTCAAGACTGCATGAATTTGTGTCTTTT | 104 nt | B |
| 17 | GGTACTAGTGGAATATCCTTTACGTTGTCTGGTG  TTGGTTGCCCAGGTTGTTGCTGAGATGTGGCG | 108 nt | A |
| 18 | GTGCAGAGAAGAAATGTATGATAAAGATATCATCATGCTTCAG  gtacctatttaaattgtttctgatatttgtgtcttcatcttcc | 128 nt | D |
| 19 | CATCTTTAATGGATCCCAATAAGTTCTTGTTACTGG  TACTTCAGAGGTATGAACTTGCCGAGGCTTTTAACAAG | 116 nt | C |
| 20 | *ggttcagcgtacaggtga*CACTAATAGAAGAAATGCTTCAGGT  CCTCATCTATATTGTGGgtaagattggcgcactatattctatc | 128 nt | E |
| 21 | GAGAGAAATCATTCACTTGCTTTGCATTGAA  CCCATGCCACACAGTGCCATTGCCAAA | 100 nt | B |
| 22 | gtactttggatactttgtagaagctctgaagttcttgcc  tgaactcccaagaataagagtggaatacagattag | 116 nt | D |
| 23 | GAAACCAGGTGTATCAGGC  CATGGAGTTTATGAACTAAAAGATGAA | 88 nt | C |
| 24 | gactaagtttttgatcagggatattcctcatc  tattttgcattaggtttctctagaacacaa | 104 nt | C |
| 25 | ATTCTGCCCTGCTTTCAGCAAAGTGATTAAC  CTTCTCAACTGTGATATCATGATGTACATTCTCAG | 108 nt | B |
| 26 | GAAGAGAAGCAACAGCTTCAAAAAGCTCCTGAAG  AAGAAGTAACATTTGACTTTTATCATAAGGCTTCAA | 112 nt | B |
| 27 | TGAATATACAAATGCTTTTGGAAAAACTC  AAAGGAATTCCCCAGTTAGAAGGCC | 96 nt | B |
| 28 | gtgacattcatctctgtctttgaactttactgg  tataagggagctactgcttttttgttaggagaaacct | 112 nt | C |
| 29 | GAAGCTGCTAGGCTACATCGCCAG  AAGATCATGGCTCAGATGTCTGCCTTACAG | 96 nt | C |
| 30 | GGGTCCTAAACGGGGTCCATCTGTTAC  TGAAAAGGAGGTGCTGACGTGCATCCT | 96 nt | D |
| 31 | GCCCTAGACCCACTTTTCATGGATCC  AGACTTGGCATATGGAACTTATACAGGAAGCT | 100 nt | C |
| 32 | GGAGAAGAATATCTTTGCCCTCTTTGCAAATC  TCTGTGCAATACTGTGATCCCCATTATTCCTTTG | 108 nt | C |
| 33 | GAGAATGCAGATGCTCTTGCTCAACTTTTGAC  CCTGGCACGGTGGATACAGACTGTTCTGGC | 104 nt | D |
| 34 | gggtttattctaacagtgccttgatg  gaaacttcttaaataaataggttaaagatgaaaact | 104 nt | E |
| 35 | TTGAAAGTGCCACCTGATGAAAGGGATCCTCGAGTC  CCCATGCTGACCTGGAGCACCTGCGCTTTCACTA | 112 nt | D |
| 36 | ccaacaaggtacatgtaactcatggc  atgccttttttcccccccacagtttattatta | 100 nt | D |
| 37 | CATAATGGTCTGAAAGCATTAATGCAGTTTGCAGTTGC  ACAGAGGATTACCTGTCCTCAGGTCCTGATACAGAAACAT | 120 nt | D |
| 38 | GATACACCATGCCTTCTGTCTATAGATCTGTTTC  ATGTTTTGgtaagtgttcagtaattttgtttaagtcactcatgttgataatt | 128 nt | C |
| 39 | GTGGGTGCTGTGTTAGCATTCC  CATCCTTGTATTGGGATGACCCTG | 88 nt | D |
| 40 | CTTTCTTTGCAGAAATTTCTCAATATACAAGTG  Ggtgagtaacaatccattagttcagtctattgt | 108 nt | D |
| 41 | GCATTGTTTTTCCACTATTTACTTGGGGTAAC  TCCGCCTGAGGAACTGCATACC | 96 nt | E |
| 42 | GTTCCTGCTCTTCCAGGAATATTGGGATAC  TGTAAGGCCCTTGCTCCAGAGgtactat | 100 nt | E |
| 43 | CTGTTTGAAGCAAAAAAACACCGTGGTC  Aggttggttttactacttaatcctttctccctcatccacaag | 112 nt | E |
| 44 | TTCCTGATGACTATAGCTGCCTCCTGAATC  AAGCTTCTCATTTCAGgtaaggagagtgtgtatatatatgtgtgtaat | 120 nt | E |
| 45 | GGGAAGAGGTTGGAGCTTGCATTTTTCACGCACTTCAC  TGTGGAGCCGGAGTCTGCATTTTCCTAAAgtgagtagtgagtgt | 124 nt | E |
| 46 | CAGAGAATGCCGAGTGGTCCTGGTTGAAGGTAAAGC  CAGAGGCTGTGCCTATCCAGCTCCTTACTTGGATGAAT | 116 nt | E |
| 47 | CATTTATCTCGTGAGCGGTATCGGAAGCTCC  ATTTGGTCTGGCAACAACACTGCATTATAGAAGAG | 108 nt | E |

Universal 5’ primer binding site: GGGTTCCCTAAGGGTTGGA

Universal 3’ primer binding site: TCTAGATTGGATCTTGCTGGCAC

Stuffer sequences are indicated by italic letters.

*UBR1* GenBank reference sequence NM_174916.2.

**Supp. Table S2:** *UBR1* probemixes (for deletion/duplication screening).

|  | **Probemix A** | | **Probemix B** | | **Probemix C** | | **Probemix D** | | **Probemix E** | |
| --- | --- | --- | --- | --- | --- | --- | --- | --- | --- | --- |
| **nt** | **Exon** | **µl** | **Exon** | **µl** | **Exon** | **µl** | **Exon** | **µl** | **Exon** | **µl** |
| **88** | - | - | - | - | 23 | 0.8 | 39 | 0.8 | - | - |
| **96** | 10 | 0.8 | 27 | 0.8 | 29 | 0.8 | 30 | 0.8 | 41 | 0.8 |
| **100** | 03 | 0.8 | 21 | 0.8 | 31 | 0.8 | 36 | 0.8 | 42 | 0.8 |
| **104** | 07 | 1.0 | 16 | 0.8 | 24 | 0.8 | 33 | 0.8 | 34 | 0.8 |
| **108** | 17 | 0.6 | 25 | 2.4 | 32 | 0.8 | 40 | 0.8 | 47 | 0.8 |
| **112** | 01 | 0.6 | 26 | 0.8 | 28 | 0.8 | 35 | 1.6 | 43 | 0.8 |
| **116** | 02 | 2.4 | 05 | 1.0 | 19 | 0.8 | 22 | 0.8 | 46 | 0.8 |
| **120** | 06 | 0.6 | 08 | 1.0 | 11 | 0.8 | 37 | 0.8 | 44 | 0.8 |
| **124** | 04 | 2.0 | 09 | 1.0 | 12 | 0.8 | 14 | 1.0 | 45 | 1.0 |
| **128** | 13 | 0.8 | 15 | 1.6 | 38 | 0.8 | 18 | 0.6 | 20 | 0.8 |
| **TE** | - | 180.8 | - | 179.6 | - | 184.0 | - | 182.4 | - | 185.2 |

**Supp. Table S3:** Prediction of pathogenicity of the *UBR1* missense variant p.Leu1597Arg (c.4790T>G) using various online prediction tools.

| **MutPred** | Probability of deleterious mutation: **0.810** |
| --- | --- |
|  | http://mutpred.mutdb.org/ |
|  | last modified 02 Feb 2014 |
|  | Li B, Krishnan VG, Mort ME, Xin F, Kamati KK, Cooper DN, Mooney SD, Radivojac P. 2009. Automated inference of molecular mechanisms of disease from amino acid substitutions. Bioinformatics 25:2744-50. |
| **PolyPhen-2 (HumVar)** | Probably damaging (**0.998**) |
|  | http://genetics.bwh.harvard.edu/pph2/ |
|  | version 2.2.2 |
|  | Adzhubei IA, Schmidt S, Peshkin L, Ramensky VE, Gerasimova A, Bork P, Kondrashov AS, Sunyaev SR. 2010. A method and server for predicting damaging missense mutations. Nat Methods 7:248-9. |
| **Mutation Assessor** | Functional impact: high (**3.875**) |
|  | http://mutationassessor.org/r3/ |
|  | Release 3 |
|  | Reva B, Antipin Y, Sander C. 2007. Determinants of protein function revealed by combinatorial entropy optimization. Genome Biol 8:R232. |
| **SIFT** | Damaging (**0**) |
|  | http://sift.jcvi.org/www/SIFT_enst_submit.html |
|  | via PROVEAN v1.1.3 |
|  | Kumar P, Henikoff S, Ng PC. 2009. Predicting the effects of coding non-synonymous variants on protein function using the SIFT algorithm. Nat Protoc 4:1073-81. |
| **Mutation Taster** | Disease causing (**0.999999990488231**) |
|  | http://www.mutationtaster.org/ |
|  | Current build: NCBI 37 / Ensembl 69 |
|  | Schwarz JM, Cooper DN, Schuelke M, Seelow D. 2014. MutationTaster2: mutation prediction for the deep-sequencing age. Nat Methods 11:361-2. |

**Supp. Figure S2:** multiple protein alignment of human UBR1 and its orthologues around position 1597.

UBR1_Hs VVRYPRKRNSLIELPDDYSCLLNQAS 1597

UBR1_Mm VVRYPRKRNSLIELPEDYSCLLNQAS 1605

UBR1_Cf IVRYPRKRNSLIELPDDYSCLLNQAS 1601

UBR1_Bt VVRYPRKRNSLIELPDDYSCLLNQAS 1590

UBR1_Gg AIRYPRKRNSLIELPEDYSCLLNQAS 1585

UBR1_Xt ALRYPRKRNTLIELPEDYSSLLNQAS 1575

UBR1_Dr MIRYPRKRNRLIDLPEDYSVLLNQAS 1531

UBR1_Dm VEPCLRPLPRLKVLCDDFSDLINSVS 1673

UBR1_Ce --VQPVRPNLLVELPEKYSQLINQVA 1904

UBR1_Sc WINNEIPGYISRVMGDEFRVTILSNG 1804

UBR2_Hs AIRYPRESNKLINLPEDYSSLINQAS 1603

Protein alignment was created using Clustal W2 (http://www.ebi.ac.uk/Tools/msa/clustalw2) [Larkin MA, Blackshields G, Brown NP, Chenna R, McGettigan PA, McWilliam H, Valentin F, Wallace IM, Wilm A, Lopez R, Thompson JD, Gibson TJ, Higgins DG. 2007. Clustal W and Clustal X version 2.0. Bioinformatics 23:2947-8] and BLOSUM62-Matrix. Numbers indicate position of affected amino acid residue. Black shading indicates identical amino acid residues; grey shading indicates similar residues (according to BLOSUM62 matrix). Hs, *Homo sapiens* (NP_777576.1); Mm, *Mus musculus* (NP_033487.2); Gg, *Gallus gallus* (XP_421165.3); Xt, *Xenopus tropicalis* (XP_002941132.2); Dr, *Danio rerio* (XP_009291507.1); Dm, *Drosophila melanogaster* (NP_573184.1); Sc, *Saccharomyces cerevisiae* (NP_011700.1); UBR2 (NP_056070.1).
